# Cupro-Sulphate of Ammonia in Neuralgia of the Fifth Pair

**Published:** 1879-12

**Authors:** Lucien Howe


### CUPRO-SULPHATE OF AMMONIA IN NEURALGIA OF THE FIFTH PAIR—BY FEREOL.

TRANSLATED FROM THE FRENCH BY LUCIEN HOWE, M. D.

THIS agent has been regarded as a sort of "specific" for epilepsy for some time—the anti-epileptic mixture of Weisman. In other neuralgias than those of the fifth pair, the writer has always been disappointed in its effects; but four such cases, in which it produced a good result, are especially mentioned. The symptoms of congestion, the epiphora and discharge from the nostril, were markedly relieved; but sometimes it produced a depressed condition of the vital forces, occasionally accompanied by nausea. The following is the method of administration:

℞ Ammoniated copper, grs. ii to iii.
Syrup of orange flower or peppermint, ʒi.
Distilled water, ℥iii.

To be taken during the day, in divided doses, about the time of meals. In spite of the metallic taste and the fetid odor of the breath, the dose of two grains in twenty-four hours ought to be continued for fifteen or twenty days.—(*Bulletin de Ther.*)—*Lyons Medicale.*

